# A Comparative Analysis of the Action Mechanisms of Cannabidiol, Cannabigerol, and Cannabinol in Human Cholangiocarcinoma Cell Lines

**DOI:** 10.3390/molecules31142446

**Published:** 2026-07-13

**Authors:** Sahaphum Laprom, Boonya Shuntawiwat, Punyabhorn Rattanacheeworn, Yamaratee Jaisin, Kiattawee Choowongkomon, Papavee Samatiwat

**Affiliations:** 1 Medical Degree Program, Faculty of Medicine, Srinakharinwirot University, Bangkok 10110, Thailand; sahaphum.laprom@g.swu.ac.th (S.L.); boonya.jan@g.swu.ac.th (B.S.); 2 Department of Pharmacology, Faculty of Medicine, Srinakharinwirot University, Papavee Samatiwat 114 Sukhumvit 23, Khlong Toei Nuea Subdistrict, Watthana District, Bangkok 10110, Thailand; punyabhorn@g.swu.ac.th (P.R.); yamaratee@g.swu.ac.th (Y.J.); 3 Center of Excellence in Medical Environmental Innovation Research (CEMEIR), Srinakharinwirot University, Bangkok 10110, Thailand; 4 Department of Biochemistry, Faculty of Science, Kasetsart University, Bangkok 10900, Thailand; fsciktc@ku.ac.th; 5 Center for Advanced Studies in Nanotechnology for Chemical, Food, and Agricultural Industries, KU Institute for Advanced Studies, Kasetsart University, Bangkok 10900, Thailand

**Keywords:** cannabidiol, cannabigerol, cannabinol, cholangiocarcinoma, anticancer, apoptosis, Ki67, proteomics, cisplatin, gefitinib

## Abstract

**Background:** Chemoresistance remains a major obstacle in managing cholangiocarcinoma (CCA). The cannabis plant contains several phytocannabinoids, including cannabidiol (CBD), cannabigerol (CBG), and cannabinol (CBN), which exhibit anticancer properties. However, to the best of our knowledge, their effects on CCA have not been previously investigated. This study aimed to explore the molecular mechanisms underlying the anticancer effects of CBD, CBG, and CBN in CCA cells. **Methods:** KKU-100 and KKU-452 cells were treated with varying concentrations of CBD, CBG, and CBN for 24 and 48 h. Cytotoxicity was assessed using the MTT assay, and half maximal inhibitory concentration (IC_50_) values were calculated. KKU 452 cells were further analyzed for apoptosis, mitochondrial membrane potential (MMP), and Ki67 expression using flow cytometry. Proteomics profiling was performed to compare the effect of these cannabinoids with those of gefitinib and cisplatin. **Results:** Monotherapy with CBD, CBG, or CBN induced dose-dependent cytotoxicity at 24 and 48 h with lower IC_50_ values than those of cisplatin and comparable efficacy to that of gefitinib. At low doses, CBD, CBG, and CBN induced early apoptosis, while higher doses triggered late apoptosis. MMP loss increased by 2.5-, 4.9-, and 1.7-fold, respectively, after 6 h. Ki67, highly expressed in KKU-452 cells (Ki67-positive ratio = 3.16 ± 0.16), was significantly reduced after the cannabinoid treatment, with Ki67-positive ratios of 0.38 ± 0.22, 0.38 ± 0.13, and 0.32 ± 0.23 for CBD, CBG, and CBN, respectively. Proteomics analysis identified 2781 proteins affected by CBD, CBG, CBN, cisplatin, and gefitinib. All three cannabinoids downregulated key upstream regulatory proteins (LARP1, TFEB, and BCR). Similar patterns of LARP1 and TFEB downregulation were also observed with cisplatin and gefitinib. CBN showed the closest similarity to cisplatin, followed by gefitinib, by targeting CDK4/6 and PCGEM1 proteins. CBD and CBG exhibited the greatest similarity to each other, also influencing MASTL expression. **Conclusions:** CBD, CBG, and CBN exhibit potential anticancer activity in CCA by suppressing proliferation, reducing Ki67 expression, and inducing apoptosis through MMP disruption. The identification of shared molecular targets, including LARP1 and TFEB, provides new mechanistic insight and supports the potential development of cannabinoid-based therapeutic strategies for cholangiocarcinoma.

## 1. Introduction

Cholangiocarcinoma (CCA) is an aggressive adenocarcinoma of the hepatobiliary system, classified into intrahepatic, perihilar, and distal subtypes [1]. The incidence and mortality rate of intrahepatic CCA have been rising, with ~50% of cases diagnosed in patients without identifiable risk factors [2]. The highest incidence of CCA has been reported in northeastern Thailand [3], where it is associated with infection by the liver fluke *Opisthorchis viverrini* [4,5], which the International Agency for Research on Cancer has classified as a group 1 carcinogen [6]. Nevertheless, the incidence of CCA in this region has decreased since 2002 [7]. In most other countries, where liver fluke infection is not endemic, CCA remains a rare malignancy. Non-fluke-related risk factors for CCA include cholangitis, cirrhosis, choledochal cyst (particularly type V), and Caroli’s disease [2]. For patients with unresectable CCA and poor prognosis, the current first-line treatment is a combination of gemcitabine and a platinum derivative [8,9,10], most notably cisplatin, which serves as a critical clinical backbone for systemic chemotherapy. Second-line chemotherapy regimens typically comprise fluoropyrimidine-based monotherapy or combinations of folinic acid, fluorouracil, and oxaliplatin (FOLFOX) [9,11,12,13]. Molecular targeted therapies have also emerged as treatment options for unresectable disease, including inhibitors of fibroblast growth factor receptor (FGFR) [14] and epidermal growth factor receptor (EGFR) [15]. Among these, gefitinib, an EGFR inhibitor, represents a key benchmark for evaluating molecular targeted interventions against aberrant receptor tyrosine kinase signaling. However, long-term treatment is frequently complicated by chemoresistance and high recurrence rates. Several studies indicate that median overall survival with first-line chemotherapy does not exceed 12.2 months [8,9,16]. Therefore, new therapeutic strategies for CCA are currently being explored both in vitro and in clinical trials. Among these, plant-derived anticancer agents represent a promising avenue for combating CCA.

Cannabis plants belong to the Cannabaceae family and include the species *Cannabis sativa*, *Cannabis ruderalis*, and *Cannabis indica.* For example, *Cannabis sativa* L. has been used as an herbal remedy since the Han Dynasty in Ancient China and in Indian Ayurvedic medicine [17]. In Thailand, cannabis has been legal since February 2022. Cannabis is the primary source of several phytocannabinoids [18,19]. Notable cannabinoids such as Δ9-tetrahydrocannabinol (THC), cannabidiol (CBD), cannabigerol (CBG), and cannabinol (CBN) have been used as antiemetics in patients receiving chemotherapy [20,21]. THC and non-THC cannabinoids have demonstrated potential anticancer effects in vitro and in vivo across a variety of cancer models, including lung, breast, prostate, hepatocellular, pancreatic, lymphatic, and skin cancers, as well as CCA [17,22,23,24]. CBD and CBG are non-psychoactive cannabinoids, with CBD present in relatively high amounts in cannabis, while CBG and CBN are considered minor cannabinoids. Previous studies have shown that CBD and CBG induce cytotoxicity in CCA cells by stimulating autophagic, apoptotic and senescence signaling pathways and inhibiting cell migration and invasion [24,25,26]. CBN, which exhibits only mild psychoactive effects, has also been shown to exert antiproliferative activity [27] and enhance chemosensitization by inhibiting the multidrug transporter ABCG2 [28,29]. The anticancer effects of these compounds are often attributed to their ability to activate cannabinoid receptors or transient receptor potential (TRP) vanilloid type-1 (TRPV1) channels, leading to promotion of cell death, inhibition of cell proliferation, and disruption of tumor-related processes including angiogenesis, migration, invasion, adhesion, and metastasis [30,31,32,33,34,35,36,37,38,39,40,41,42,43,44]. However, the molecular mechanisms underlying the anticancer effects of CBD, CBG, and CBN in CCA have not yet been elucidated. To resolve these mechanisms, we investigated their effects through apoptosis analysis, mitochondrial membrane potential assessment, Ki67 proliferation analysis, and quantitative high-throughput proteomic profiling. We hypothesized that the non-psychoactive cannabinoids CBD, CBG, and CBN induce cytotoxicity and apoptosis in CCA cells by disrupting mitochondrial membrane potential and suppressing cell proliferation, mediated through the modulation of candidate upstream regulators (such as LARP1 and TFEB) that converge with pathways targeted by standard clinical chemotherapeutic benchmarks.

## 2. Results

### 2.1. Cytotoxic Activity of CBD, CBG, and CBN

**Figure 1:**
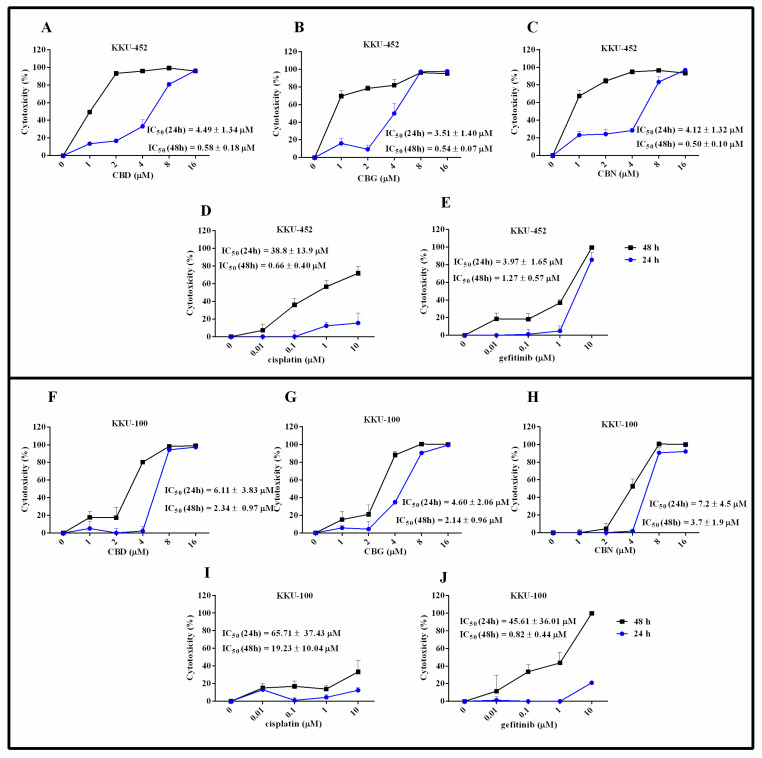
Cytotoxic effects of cannabinoids and controls in CCA cells. Cell viability was assessed by MTT assay after 24 and 48 h of treatment. CBD, CBG, CBN, cisplatin, and gefitinib induced cytotoxicity in KKU-452 (**A**–**E**) and KKU-100 (**F**–**J**) cells in a dose- and time-dependent manner. Values represent the mean ± SE from three independent experiments performed in quadruplicate.

The cytotoxic effects of cannabinoids (CBD, CBG, and CBN), cisplatin, and gefitinib on cholangiocarcinoma cells were evaluated using the MTT assay. All three cannabinoids suppressed CCA cell proliferation in a concentration and time-dependent manner. CBD, CBG, CBN, cisplatin, and gefitinib induced cytotoxicity both in KKU-452 (Figure 1A–E) and KKU-100 (Figure 1F–J) cells after 24 and 48 h of treatment. At 16 μM, CBD, CBG, and CBN produced maximal growth inhibition that persisted through 48 h. In KKU-452 cells, the half-maximal inhibitory concentration (IC_50_) values for CBD, CBG, and CBN were 4.49 ± 1.34, 3.51 ± 1.40, and 4.12 ± 1.32 μM at 24 h, and 0.58 ± 0.18, 0.54 ± 0.07, and 0.50 ± 0.10 μM at 48 h, respectively (Table 1). Similar dose- and time-dependent response patterns were observed in KKU-100 cells, with IC_50_ values of 6.11 ± 3.83, 4.60 ± 2.06, and 7.20 ± 4.50 μM at 24 h, and 2.34 ± 0.97, 2.14 ± 0.96, and 3.70 ± 1.90 μM at 48 h, respectively (Table 1). These data indicate that KKU-452 cells are more sensitive to CBD, CBG, and CBN than KKU-100 cells. The results also demonstrate that CBD, CBG, and CBN exert potent cytotoxic effects that surpass those of cisplatin (a conventional chemotherapy drug) and gefitinib (an EGFR-targeted therapy) in both CCA cell lines. Moreover, CCA cells displayed resistance to cisplatin, whereas gefitinib showed a good response, especially in KKU-100 cells at 48 h.

Half-maximal inhibitory concentration (IC_50_) values of CBD, CBG, and CBN were determined in comparison with cisplatin (conventional chemotherapy) and gefitinib (EGFR-targeted therapy) after 24 and 48 h of treatment in KKU-100 and KKU-452 cells. IC_50_ represents the minimum concentration of the compound required to reduce the cell viability by 50% compared with the untreated control. Values represent the mean ± SE from three independent experiments performed in quadruplicate. CBD, cannabidiol; CBG, cannabigerol; CBN, cannabinol.

### 2.2. Induction of Apoptosis by CBD, CBG, and CBN

**Figure 2:**
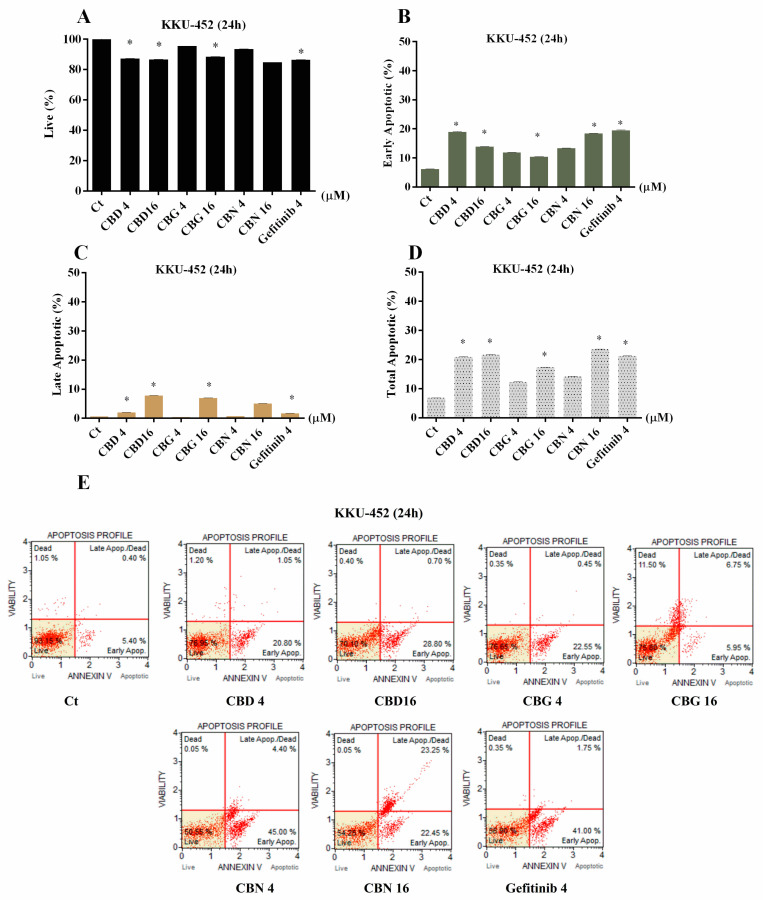
Apoptosis-inducing effects of CBD, CBG, and CBN in KKU-452 cells. (**A**), Percentages of live cells (**B**), early apoptotic cells (**C**), late apoptotic cells and (**D**) total apoptotic cells following 24 h treatment. Bars represent the mean ± SD from six independent experiments. (**E**) Representative flow cytometry plots obtained using annexin V and dead cell kit staining. The red lines divide each plot into four quadrants representing live, early apoptotic, late apoptotic, and dead cells. * *p* < 0.05 vs. control (Ct). CBD, cannabidiol; CBG, cannabigerol; CBN, cannabinol.

Because KKU-452 cells showed greater sensitivity to cannabinoids in the cytotoxic assay, they were selected for apoptosis analysis at 24 h. Gefitinib was included as a positive control for apoptosis induction. Following treatment, cells were stained with Annexin V and 7-AAD and analyzed using flow cytometry. CBD, CBG, and gefitinib significantly reduced the proportion of viable cells compared with the untreated control (Figure 2A). Gefitinib and high doses of CBD, CBG, and CBN markedly increased the proportion of early apoptotic cells (Figure 2B). Gefitinib, IC_50_ concentrations of CBD, and high concentrations of CBD and CBG also significantly increased late apoptotic cells, while high-dose CBN showed a trend toward inducing late apoptosis (Figure 2C). Both IC_50_ and high-dose CBD strongly promoted total apoptotic cell death; high-dose CBG and CBN also effectively triggered cell death (Figure 2D). Representative flow cytometry plots showing live, early apoptotic, late apoptotic, and dead cells are presented in Figure 2E. Collectively, these data suggest that CBD was more effective than CBG and CBN in inducing apoptosis at 24 h post-treatment. Overall, the phytocannabinoids induced a concentration-dependent cytotoxicity in both cell lines; however, no significant differences were observed between the control group and those treated with either 4 µM CBG or 16 µM CBN.

### 2.3. Alteration of MMP by CBD, CBG, and CBN

**Figure 3:**
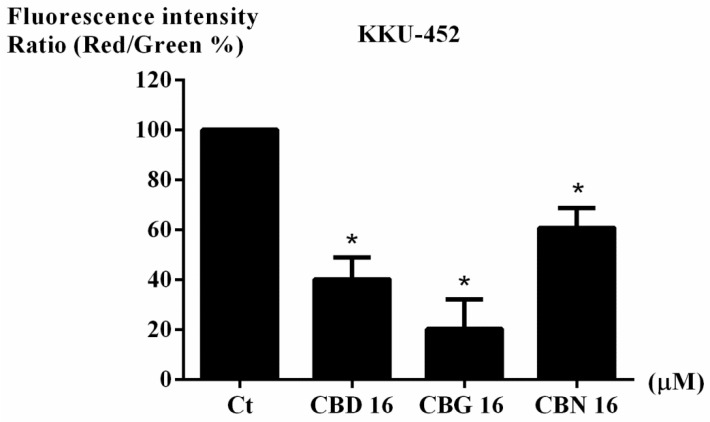
Effects of CBD, CBG, and CBN on mitochondrial membrane potential (MMP, Δψm) in KKU-452 cells. KKU-452 cells were treated with 16 µM of CBD, CBG, or CBN for 6 h and stained with JC-1 fluorescent dye. MMP was quantified by calculating the red/green fluorescence intensity ratio and normalizing it to the control (Ct) as a percentage. Data represents SD from three independent experiments. * *p* < 0.05 vs. control. CBD, cannabidiol; CBG, cannabigerol; CBN, cannabinol.

Changes in MMP reflect alterations in mitochondrial function and can indicate mitochondrial dysfunction leading to cell death. To investigate the effects of cannabinoids on MMP, KKU-452 cells were treated with 16 µM of CBD, CBG, or CBN for 6 h, and then stained with JC-1 dye. Compared with the control group, the red/green fluorescence intensity ratio (a measure of MMP) was significantly decreased by 2.5-, 4.9-, and 1.7-fold in cells treated with CBD, CBG, and CBN, respectively (Figure 3). These findings indicate that CBD, CBG, and CBN induce apoptosis in KKU-452 cells, at least in part, by disrupting the MMP.

### 2.4. CBD, CBG, and CBN Suppressed Ki67 Expression

**Figure 4:**
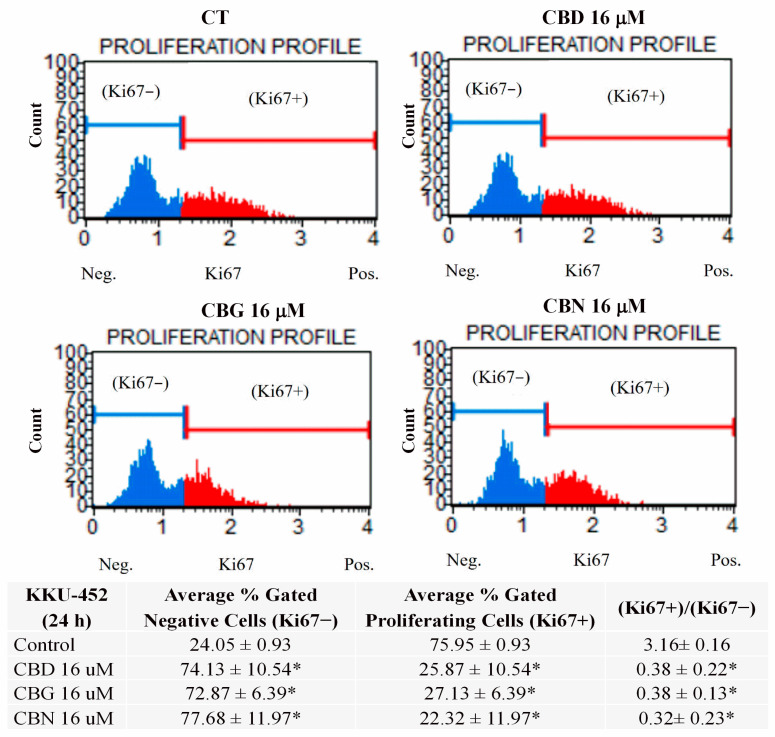
Effect of CBD, CBG, and CBN on Ki67 expression in KKU-452 cells. KKU-452 cells were treated with 16 µM of CBD, CBG, or CBN for 24 h. Representative histogram plots show two cell populations: Ki67-negative (blue) and Ki67-positive (red), as determined using the Muse Ki67 assay. The accompanying table presents the mean percentages of gated Ki67-negative and Ki67-positive cells and the Ki67+/Ki67− ratio. Data are expressed as mean ± SD from three independent experiments. * *p* < 0.05 vs. control (Ct), CBD, cannabidiol; CBG, cannabigerol; CBN, cannabinol.

Ki67 is a well-established marker of cell proliferation, highly expressed in cancer cells but absent in normal cells [31]. Given that the Ki-67 protein serves as an essential biomarker for cellular proliferation and active cell cycle progression [45]. The effects of CBD, CBG, and CBN on Ki67 nuclear protein expression were assessed in KKU-452 cells using the Muse^®^ Ki67 assay after 24 h of treatment. KKU-452 cells exhibited high baseline Ki67 expression, which was markedly reduced following exposure to 16 µM CBD, CBG, or CBN (Figure 4, red histogram). Statistical analysis of the gated cell populations revealed a significant increase in the proportion of Ki67− cells and a corresponding decrease in Ki67+ proliferating cells in the treated groups compared with the control. These findings indicate that CBD, CBG, and CBN suppress nuclear Ki67 expression, thereby reducing proliferation across all active phases of the cell cycle (G1, S, G2, and M phases) and potentially impairing survival.

### 2.5. Effect of CBD, CBG, and CBN on Differential Protein Expression

**Figure 5:**
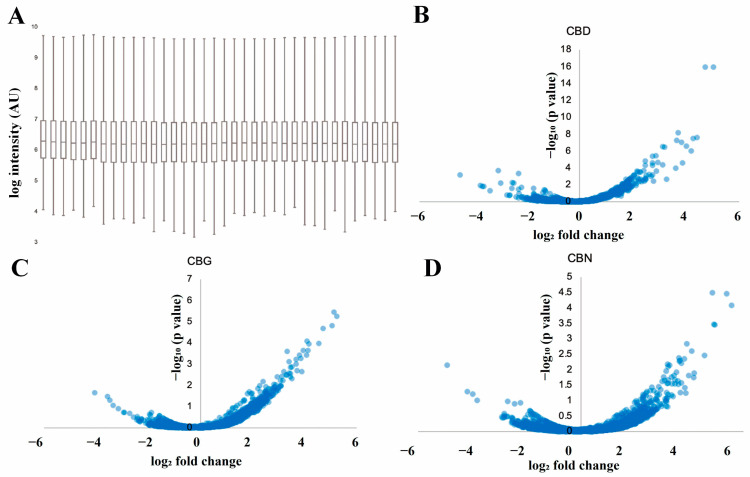
Quantitative proteomics analysis of differential protein expression in KKU-452 cells following treatment with CBD, CBG, and CBN. (**A**) Log-intensity distribution of protein quantification across all samples; (**B**–**D**) Volcano plots showing the log2 fold change (*x*-axis) versus the −log10 *p*-value (*y*-axis) for upregulated and downregulated proteins in KKU-452 cells treated with 16 µM of CBD, CBG, and CBN, respectively, compared with control.

To comprehensively examine global protein expression patterns, proteomics analysis was performed. Standardizing the initial protein concentrations across all samples is essential for accurate quantification. After normalization, the total peptide intensities for each experimental group, including technical replicates, were evaluated, revealing no significant differences among the groups (*p* > 0.99; Figure 5A).

In total, 4539 proteins were identified, of which 2781 proteins contained >2 unique peptides (Supplementary File S1). The differentially expressed proteins identified in CBD-, CBG-, and CBN-treated groups (each with >2 unique peptides) are shown in Figure 5B–D, respectively (Supplementary File S2).

### 2.6. Comparative Analysis of Biochemical Pathways and Upstream Regulators

**Figure 6:**
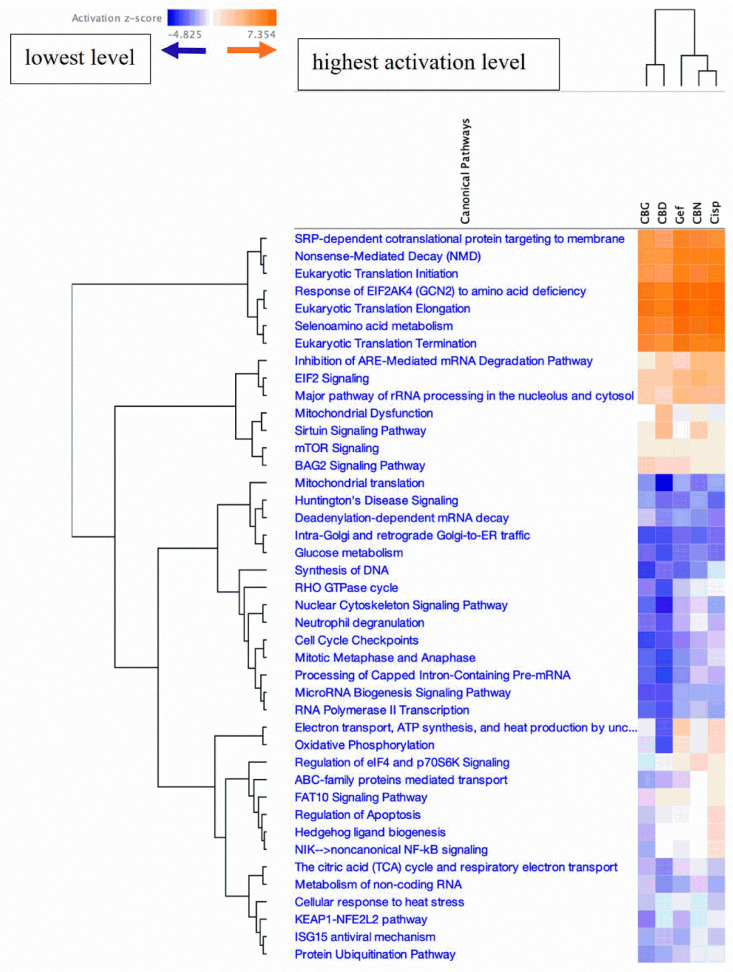
Heatmap analysis of differential biochemical pathway activation in KKU-452 cells treated with CBD, CBG, CBN, cisplatin (Cisp), and gefitinib (Gef). Pathway activation *z*-scores are color-coded from blue (low activation) to orange (high activation). Two main clusters were observed: the first, comprising CBN, cisplatin, and gefitinib; and the second, comprising CBD and CBG.

Global biochemical pathway alterations induced by CBD, CBG, and CBN in KKU-452 cells were analyzed using Ingenuity Pathway Analysis (IPA) (Figure 6; Supplementary File S3). To place the cannabinoid-induced responses into a broader biological context, the pathway activation profiles were compared with those of two reference anticancer drugs, cisplatin and gefitinib. This comparison was intended to identify similarities and differences in predicted downstream pathway responses rather than to infer shared molecular mechanisms or direct drug–target interactions.

Hierarchical clustering based on IPA-derived pathway activation *z*-scores identified two major clusters of compounds. One cluster included CBN together with cisplatin and gefitinib, whereas the second cluster comprised CBD and CBG. Four levels of pathway activation or inhibition were distinguished according to the predicted pathway activation scores. In particular, pathways assigned to inactivation level 4 displayed opposite activation patterns between the two clusters, indicating that these treatments elicited distinct profiles of downstream biological responses. Because the clustering analysis was performed using pathway activation *z*-scores, the proximity of compounds within the dendrogram reflects similarities in predicted pathway-level responses rather than mechanistic equivalence or shared primary molecular targets. Pharmacologically distinct anticancer agents may converge on common stress-response pathways, including apoptosis, mitochondrial dysfunction, DNA damage responses, cell-cycle arrest, and metabolic reprogramming, despite acting through different upstream molecular events.

**Figure 7:**
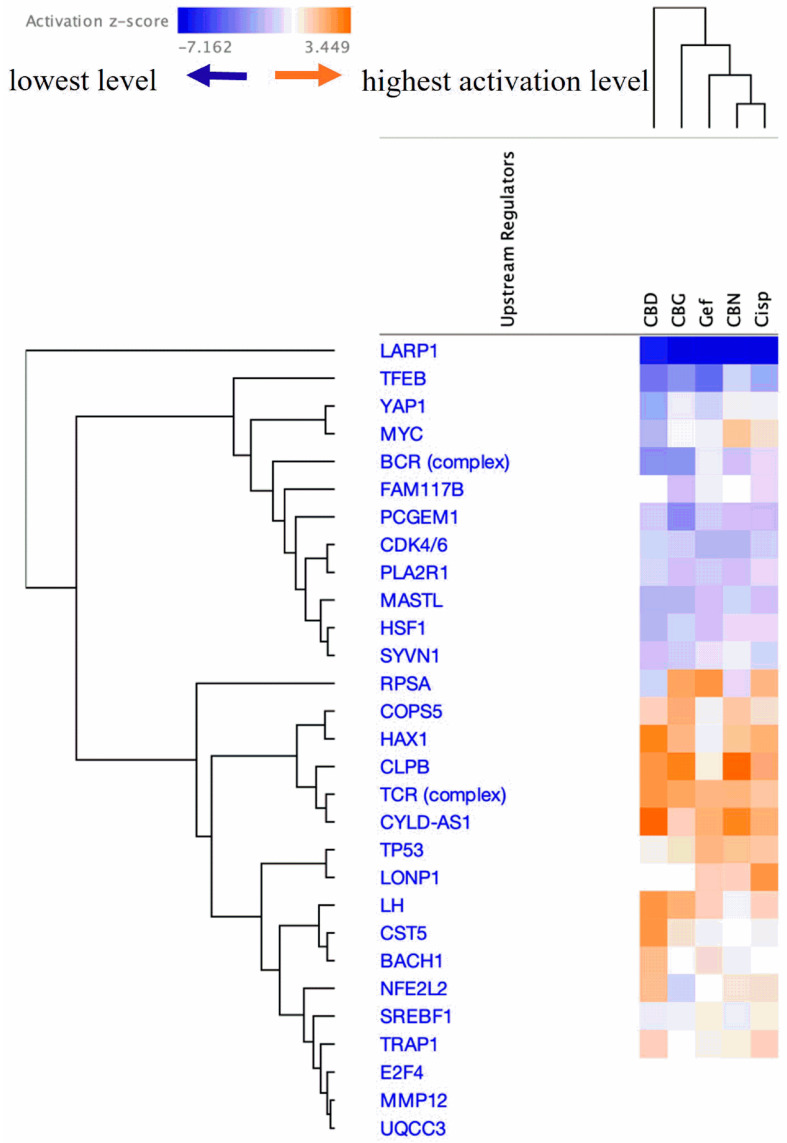
Prediction of upstream protein regulators in KKU-452 cells following treatment with CBD, CBG, CBN, cisplatin, and gefitinib. Predicted activation or inhibition of upstream protein regulators is represented by positive or negative *z*-scores, respectively. The heatmap highlights the top six proteins most impacted by these treatments.

**Figure 8:**
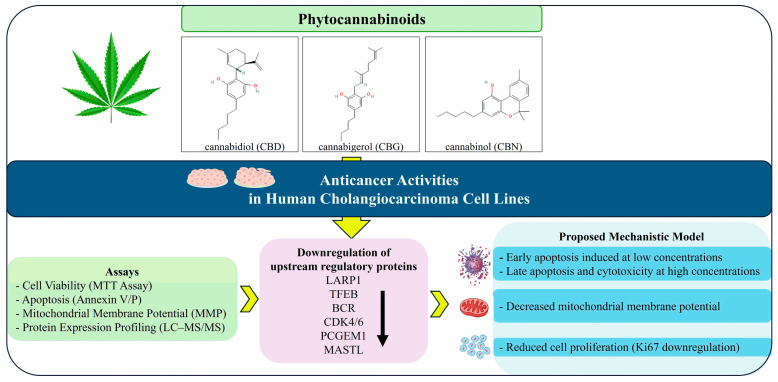
Proposed overview of the anticancer effects of the phytocannabinoids cannabidiol (CBD), cannabigerol (CBG), and cannabinol (CBN) in cholangiocarcinoma (KKU-452) cells. All three cannabinoids reduced cell viability and proliferation, decreased nuclear Ki67 expression, disrupted mitochondrial membrane potential (MMP), and induced apoptosis. Comparative proteomic analysis coupled with Ingenuity Pathway Analysis (IPA) identified common candidate upstream regulators, including LARP1, TFEB, and BCR, while pathway clustering revealed that CBD and CBG shared similar pathway-response profiles, whereas CBN exhibited downstream pathway responses more similar to those of cisplatin and gefitinib. These predicted regulatory pathways represent candidate molecular mediators requiring further functional validation.

To further investigate the biological processes underlying these pathway alterations, upstream regulator prediction analysis was performed using IPA (Figure 7; Supplementary File S4). The six highest-ranked predicted upstream regulators for each treatment are summarized in Table 2 together with their activation *z*-scores, adjusted *p*-values, and fold changes. All three cannabinoids were predicted to inhibit several regulators associated with tumor progression, including La-related protein 1 (LARP1), transcription factor EB (TFEB), and the B-cell receptor (BCR) signaling complex. Similar inhibition of LARP1 and TFEB was also predicted following cisplatin and gefitinib treatment, suggesting convergence of downstream regulatory responses despite their distinct pharmacological targets. CBN clustered more closely with cisplatin and gefitinib at the pathway-response level and was associated with predicted regulation of cyclin-dependent kinases 4 and 6 (CDK4/6) and prostate cancer gene expression marker 1 (PCGEM1). In contrast, CBD and CBG formed a separate cluster and were associated with predicted regulation of microtubule-associated serine/threonine-protein kinase-like (MASTL). These observations indicate that although the cannabinoids produce overlapping downstream pathway responses with established anticancer agents, they may engage different upstream molecular events. Therefore, the present proteomic findings should be interpreted as identifying candidate regulatory pathways and potential molecular mediators that warrant further functional validation rather than establishing definitive mechanisms of action. A schematic summary of the proposed anticancer mechanisms of CBD, CBG, and CBN in KKU-452 cells is presented in Figure 8.

## 3. Discussion

Cannabinoids have attracted increasing interest as potential anticancer agents because of their ability to modulate multiple cellular processes involved in tumor progression [27,46,47,48]. However, comparative information regarding the biological activities and molecular responses of cannabidiol (CBD), cannabigerol (CBG), and cannabinol (CBN) in cholangiocarcinoma remains limited. In the present study, all three phytocannabinoids significantly reduced cell viability, inhibited cell proliferation, decreased nuclear Ki67 expression, disrupted mitochondrial membrane potential (MMP), and induced apoptosis in KKU-452 cells. Although CBD, CBG, and CBN exhibited comparable antiproliferative activities, comparative proteomic analysis revealed both shared and distinct pathway-response profiles, suggesting that structurally related cannabinoids may converge on common anticancer phenotypes while engaging partially different regulatory networks.

Although CCAs respond well to gefitinib due to overexpression of the EGFR signaling pathway, which contributes to carcinogenesis and cancer invasion [15,49,50], no targeted therapies for CCA are currently approved. A previous study reported that CBG exerts a stronger cytotoxic effect than 5-fluorouracil [51] CBD was also shown to enhance the chemosensitization effect of doxorubicin in triple-negative breast cancer (TNBC) [52] and improve IC_50_ values for both doxorubicin and gemcitabine [52,53]. A previous study indicated CBD may interact with chemotherapeutic drugs in antagonistic, additive, or synergistic ways, depending on the specific agent [54,55]. CBD and CBN have also been shown to reduce lung cancer growth in mice [23,30]. In this study, no significant differences were observed among CBD, CBG, and CBN in terms of cytotoxicity against CCA cell lines, with cytotoxic effects increasing in a concentration- and time-dependent manner. However, some studies indicate that CBD exhibits greater cytotoxic activity against cancer cell lines compared with other cannabinoids [30,56,57,58,59,60,61,62].

It is critical to address the translational relevance of the phytocannabinoid concentrations utilized in this study. Our in vitro investigation revealed IC_50_ values ranging from 0.5 to 7 µM, with experimental treatment concentrations extending up to 16 µM. Another report, cannabidiol (CBD) has been shown to inhibit CCA cell proliferation with IC_50_ values ranging from 19.66 to 21.05 µM in gemcitabine-resistant models [25]. Conversely, pharmacokinetic studies in healthy human volunteers show peak plasma concentrations (Cmax) for orally administered CBD that are orders of magnitude lower. For instance, standard oral doses of CBD typically result in mean Cmax values around 389.17 ± 153.23 ng/mL (approximately 1.24 µM) even under high-dose regimens [63]. Other formulations, including self-nanoemulsifying drug delivery systems (SEDDS), have achieved significantly enhanced absorption, yet these still result in Cmax values far below the 16–20 µM range required for potent anticancer effects in vitro [64,65]. To circumvent these pharmacokinetic limitations and achieve therapeutic concentrations within tumor tissues without systemic toxicity, alternative drug delivery strategies must be considered.

The loss of mitochondrial membrane potential observed following cannabinoid treatment is consistent with activation of the intrinsic apoptotic pathway. Mitochondrial depolarization represents an early event preceding cytochrome c release and caspase activation, ultimately leading to programmed cell death [66]. A previous study has similarly demonstrated that CBD induces mitochondrial dysfunction, oxidative stress, and apoptosis by regulating XIAP/Smac in gastric cancer cells [67]. Our findings extend these observations to cholangiocarcinoma and further demonstrate that mitochondrial dysfunction occurred concurrently with reduced Ki67 expression, indicating that the suppression of proliferation and the induction of apoptosis are coordinated cellular responses following cannabinoid exposure.

Numerous preclinical studies have shown that cannabinoids inhibit proliferation and induce apoptosis by promoting DNA fragmentation, causing cell-cycle arrest, downregulating cell-cycle-promoting factors (cyclin D, cyclin E, CDK2, CDK4, RB, and E2F1) and Bcl-2, inhibiting ATP production, increasing ROS generation, upregulating Bax, caspase3/7, PARP cleavage, ceramide synthesis, Ca^2+^ influx, and COX-2 expression [68]. Moreover, cannabinoids suppress migration and invasion by inhibiting MMP1, MMP2, MMP4, MMP9, VEGFR, and PDGF-AA [68]. In addition to apoptosis, CBD, CBG, and CBN have been reported to modulate autophagy and mitophagy in cancer cells, processes that balance cellular survival and apoptosis to eliminate malignant cells [69,70]. In breast cancer cells, CBD alters Beclin-1 and LC3-II autophagy proteins and increases cleaved PARP, cytochrome C, and caspase-8, triggering apoptosis [71,72,73]. Mitophagy, the selective autophagy of mitochondria, is essential for maintaining mitochondrial quality and structure, and identifying its regulators may provide key therapeutic opportunities [74]. Furthermore, cannabinoid receptors (CBs), which are also expressed in mitochondria, modulate mitochondrial function and morphology and are implicated in the regulation of autophagy and mitophagy in cancer cells [75,76].

Overexpression of CB1 or CB2 has been reported to increase Ki-67 expression in breast cancer cells, promoting cancer growth through activation of the IGF-1R/AKT/GSK-3β signaling pathway [77]. Our data demonstrated a significant reduction in Ki67 expression in CCA cells following treatment with CBD, CBG, and CBN. Consistently, a cannabinoid formulation containing THC and CBD downregulated Ki67 in human pancreatic ductal adenocarcinoma xenograft models [78]. However, previous studies have reported that CBD did not affect Ki67 levels, whereas THC reduced Ki67 through the orphan cannabinoid receptor GPR55 in glioblastoma [79]. Notably, a strong association has also been observed between Ki67 expression and the antiapoptotic protein Bcl-2 in colorectal cancer, which facilitates cancer and malignant transformation [80]. Therefore, the suppression of Ki67 may reflect apoptosis induction in CCA cells. These findings indicate that cannabinoids may act, at least in part, by targeting Ki67 in CCA treatment.

Proteomic profiling provided additional insight into the biological processes associated with cannabinoid treatment. IPA predicted that CBD, CBG, and CBN were associated with the inhibition of several upstream regulators, particularly LARP1, TFEB, and the B-cell receptor (BCR) signaling complex. These proteins have previously been implicated in mTOR-dependent protein translation, autophagy, lysosomal biogenesis, and tumor progression [81,82,83,84]. Downregulation of these regulatory networks is therefore consistent with the observed reduction in proliferative capacity and increased apoptosis. Nevertheless, these predicted regulators should be interpreted as candidate molecular mediators identified through system-level proteomic analysis rather than confirmed molecular targets.

Integrating the experimental and proteomic findings, a working biological model can be proposed in which cannabinoid treatment is associated with the predicted inhibition of LARP1 and TFEB, resulting in suppression of proliferative signaling, impairment of mitochondrial homeostasis, and induction of apoptosis, ultimately leading to reduced Ki67 expression and decreased cholangiocarcinoma cell survival. Importantly, this model represents a hypothesis generated from quantitative proteomic profiling and bioinformatic prediction rather than direct mechanistic evidence. Functional studies will therefore be required to determine the causal contribution of these regulators to cannabinoid-induced cytotoxicity.

Comparative pathway analysis further demonstrated that CBD and CBG displayed highly similar pathway-response profiles, whereas CBN clustered with cisplatin and gefitinib based on IPA-derived pathway activation *z*-scores. This observation should not be interpreted as evidence that these compounds share identical mechanisms of action. Instead, the clustering reflects similarities in predicted downstream biological responses, indicating pathway-level convergence rather than mechanistic equivalence. Pharmacologically distinct anticancer agents commonly activate conserved stress-response programs, including apoptosis, mitochondrial dysfunction, DNA damage signaling, cell-cycle arrest, and metabolic reprogramming, despite acting through different primary molecular targets. Accordingly, the clustering observed in the present study suggests that CBN elicits downstream cellular responses comparable to those induced by cisplatin and gefitinib without implying common upstream drug–target interactions.

The biological basis underlying the distinct clustering pattern of CBN compared with CBD and CBG remains to be fully elucidated. One possible explanation is that CBN possesses pharmacological properties that differ from those of CBD and CBG despite their structural similarity. Unlike CBD, which exhibits relatively low affinity for the classical cannabinoid receptors, CBN has been reported to act as a weak partial agonist at both CB1 and CB2 receptors, potentially leading to the differential modulation of downstream signaling pathways. In addition, CBN possesses a fully aromatic tricyclic structure formed through oxidative conversion of Δ9-tetrahydrocannabinol (THC), whereas CBD and CBG have more flexible open-chain structures. These structural differences may influence receptor interactions, intracellular target engagement, membrane permeability, and metabolic stability, thereby contributing to the distinct pathway-response profile observed in the present proteomic analysis. Although this hypothesis is biologically plausible, further pharmacological and mechanistic studies will be required to determine whether receptor selectivity or structural characteristics directly account for the unique proteomic signature of CBN.

Among the predicted upstream regulators, LARP1, TFEB, CDK4/6, MASTL, and PCGEM1 are all recognized contributors to tumor progression and therapeutic resistance. LARP1 regulates the mTOR-dependent translation of growth-promoting transcripts [85,86], whereas TFEB controls lysosomal biogenesis and autophagy—processes that frequently support cancer-cell survival under metabolic stress [87,88,89,90]. CDK4/6 is a central regulator of G1/S cell-cycle progression [91], while MASTL promotes mitotic progression and resistance to chemotherapy [92,93,94]. Although these proteins have established oncogenic functions, the present study does not demonstrate that they directly mediate cannabinoid activity. Rather, they should be regarded as candidate regulators that warrant further mechanistic investigation using targeted molecular approaches.

The partial overlap in predicted downstream pathway responses between cannabinoids and the reference drugs also raises the possibility that combination therapy may enhance therapeutic efficacy. Previous studies have shown that cannabinoids can produce additive or synergistic effects when combined with conventional anticancer agents in selected tumor models [54,55]. In the present study, common predicted regulators, including LARP1, TFEB, and CDK4/6, were identified across cannabinoid-treated and reference-drug-treated cells, suggesting that these pathways may represent rational targets for combination therapy. Future studies should therefore investigate whether phytocannabinoids enhance the activity of cisplatin, gefitinib, or clinically approved CDK4/6 inhibitors through synergistic modulation of shared downstream signaling pathways.

Several limitations should be acknowledged. First, the upstream regulators identified by IPA were computationally predicted from quantitative proteomic data and were not experimentally validated. Consequently, these proteins should be regarded as candidate molecular mediators within a hypothesis-generating framework rather than definitive mechanistic evidence. Second, functional studies—including gene knockdown, rescue experiments, pharmacological inhibition, and in vivo validation—were beyond the scope of the present work but will be essential for establishing causal relationships between the predicted regulators and cannabinoid-induced anticancer activity. Finally, although the concentrations used in vitro produced robust biological responses, additional pharmacokinetic and translational studies will be required to determine whether therapeutically relevant intratumoral concentrations can be achieved in vivo.

Overall, the present study demonstrates that CBD, CBG, and CBN produce comparable antiproliferative and pro-apoptotic effects in cholangiocarcinoma cells while exhibiting both shared and distinct pathway-response signatures. These findings provide a systems-level framework for understanding cannabinoid-induced biological responses and identify candidate signaling pathways for future mechanistic studies and rational combination strategies with established anticancer therapies.

## 4. Materials and Methods

### 4.1. Cell Cultures

Two human cholangiocarcinoma (CCA) cell lines, KKU-100 (JCRB1568) [95] and KKU-452 (JCRB1772) [96], were used in this study. Both cell lines were established at the Cholangiocarcinoma Research Institute, Faculty of Medicine, Khon Kaen University (Khon Kaen, Thailand) and are available from the Japanese Cancer Research Resources Bank (Ibaraki City, Osaka, Japan). The KKU-100 cell line has been previously described [95], and the KKU-452 cell line was established from tumor tissue resected from a CCA patient, as reported in a previous study [96]. The KKU-452 cell line was specifically selected for subsequent downstream mechanistic investigations due to its highly aggressive and proliferative phenotype, which represents a robust clinically relevant in vitro model of advanced cholangiocarcinoma compared to the slower-growing KKU-100 line. Furthermore, preliminary screening confirmed that KKU-452 cells exhibit a more suitable baseline expression profile of our target marker genes, making it an ideal candidate to study therapeutic responses. Cells were routinely cultured in Ham’s F12 medium (Gibco, Grand Island, NY, USA; Thermo Fisher Scientific, Inc., Waltham, MA, USA) supplemented with 12.5 mM *N*-2-hydroxyethylpiperazine-*N*′-2-ethanesulfonic acid (HEPES, pH 7.3) (Sigma-Aldrich, St. Louis, MO, USA), 100 U/mL penicillin, 50 μg/mL gentamicin, and 10% fetal bovine serum (Gibco; Thermo Fisher Scientific, Inc.). Cultures were maintained at 37 °C in a humidified incubator under 5% CO_2,_ as described previously [97]. Cells were sub-cultured every 2–3 days before reaching confluence using 0.25% (*v*/*v*) trypsin–EDTA (Gibco; Thermo Fisher Scientific, Inc.), and the medium was replaced after overnight incubation. Cells were passaged at approximately 70–80% confluence. For all experiments, the cells were utilized within 10 passages after thawing to ensure phenotypic stability.

### 4.2. MTT Assay

KKU-100 and KKU-452 cells were seeded into 96-well plates at a density of 7500 cells/well and allowed to attach overnight. Cells were then treated in quadruplicate with either 0.1% dimethyl sulfoxide (DMSO, Slidell, LA, USA; Sigma Chemical, Burlington, MA, USA) as an untreated control or with 1–16 μM of CBD, CBG, or CBN (Sigma Chemical), 1–100 μM of cisplatin, or 1–100 μM of gefitinib for 24 or 48 h. DMSO was selected as the vehicle control owing to the highly hydrophobic nature of the phytocannabinoids. To preclude any solvent-induced cytotoxicity, the final concentration of DMSO in all experimental groups was maintained strictly below 0.1% (*v*/*v*), a level widely recognized as non-toxic. Following treatment, cell viability was assessed using the MTT (3-(4,5-dimethylthiazol-2-yl)-2,5-diphenyltetrazolium bromide) assay. MTT reagent (Sigma Chemical) was added at a 1:100 dilution and incubated for 2 h 30 min at 37 °C. The medium was then removed, and the resulting formazan crystals were suspended in DMSO. Absorbance was read at 570 nm using a SpectraMaxM2 microplate reader (Molecular Devices, LLC, San Jose, CA, USA). The experiments were performed independently in triplicate. Percentage cytotoxicity was calculated as 100-(absorbance of treated group/absorbance of untreated control × 100). Half-maximal inhibitory concentration (IC_50_) values were determined by nonlinear curve fitting.

### 4.3. Apoptosis

Live cells and cells at various stages of apoptosis (early and late) or cell death were assessed using the Muse^®^ Annexin V & Dead Cell Kit (MCH100105; Merk Millipore, Darmstadt, Germany), as previously described [15]. Briefly, KKU-452 cells were seeded into 6-well plates at a density of 2.5 × 10^5^ cells/well and allowed to adhere overnight. Cells were then treated for 24 h with either 0.1% dimethyl sulfoxide (DMSO) as an untreated control, CBD, CBG, or CBN at 4 or 16 μM, or gefitinib at 4 μM. Following treatment, cells were harvested and stained with Annexin V and 7-AAD dead cell reagent by adding 100 μL of the dye mixture to each single-cell suspension and incubating for 20 min at room temperature (25 °C) in the dark. The total cell population was analyzed using flow cytometry (Guava^®^ Muse^®^ cell analyzer; Guava Easy Cyte HT, Millipore, Bedford, MA, USA). The proportions of live, early apoptotic, late apoptotic, and dead cells were compared to those in the untreated cell group.

### 4.4. JC-1 Mitochondrial Membrane Potential Analysis

JC-1 is a fluorescent probe that assesses mitochondrial membrane potential by measuring the ratio of red fluorescence from mitochondrial aggregates to green fluorescence from monomeric dye in depolarized mitochondria [98]. For MMP measurement, KKU-452 cells were seeded into black 96-well plates at a density of 1 × 10^5^ cells/well and cultured overnight at 37 °C in a humidified 5% CO_2_ atmosphere. Cells were then treated with 0.1% DMSO as an untreated control or with 16 μM of CBD, CBG, or CBN. Based on preliminary time-course kinetics optimization, a treatment duration of 6 h was explicitly selected to evaluate the early onset of mitochondrial dysfunction and membrane potential disruption prior to the initiation of late-stage apoptotic cell detachment. Following the 6 h incubation, cells were washed with PBS and stained with 5 mg/mL of JC-1 (ab141387, Abcam, Cambridge, UK) according to the manufacturer’s instructions. Fluorescence of JC-1 monomers (green) and aggregates (red) was measured using a Biotek Synergy HT microplate reader (Winooski, VT, USA). The red/green fluorescence intensity ratio, reflecting MMP, was calculated as the ratio of red fluorescence intensity to green fluorescence intensity and normalized to the untreated control group.

### 4.5. Ki67 Expression Assay

The proportion of proliferating versus nonproliferating cells, based on Ki67 expression, was determined using the Muse^®^ Ki67 Proliferation kit (MCH100114; Merk Millipore, Germany). KKU-452 cells were seeded into 6-well plates at a density of 2.5 × 10^5^ cells/well and allowed to adhere overnight. Cells were then treated with either 0.1% dimethyl sulfoxide (DMSO) as an untreated control or 16 μM of CBD, CBG, or CBN for 24 h. Following treatment, cells were harvested to obtain a single-cell suspension. To ensure robust antibody intracellular staining, cells were fixed with 4% paraformaldehyde for 15 min and subsequently permeabilized using 0.1% Triton X-100 for 10 min. Following these preparation steps, 150 μL of Ki67 antibody solution was added to each single-cell suspension and incubated for 30 min at room temperature. The percentages of nonproliferating (Ki67-negative) and proliferating (Ki67-positive) cells were quantified using a Muse^®^ cell analyzer (Guava EasyCyte HT, Millipore, Bedford, MA, USA).

### 4.6. Sample Preparation for Proteomics Analysis

To investigate the protein expression profile of the cells treated with compounds, whole-cell lysates were prepared using lysis buffer (1% Triton X-100, 2 mM TCEP, 10 mM NaCl in 50 mM HEPES-KOH, pH 8.0) supplemented with a protease and phosphatase inhibitor cocktail [99]. Total cellular proteins were collected by centrifugation at 16,000× *g* for 30 min and precipitated with 20% methanol/acetone (1:9, *v*/*v*) at −20 °C for 48 h. Samples were subsequently centrifuged at 10,000× *g* for 5 min, and the supernatant was discarded. After precipitation, protein pellets were reconstituted in 0.5% RapidGest SF (Waters, Wilmslow, UK) in 5 mM ammonium bicarbonate and 5 mM NaCl. Protein concentrations were determined using a bicinchoninic acid (BCA) assay kit (Pierce, New York, NY, USA), with bovine serum albumin as the standard. For protein digestion and clean-up, 20 µg of protein was subjected to tryptic digestion. Reduction of the sulfhydryl bonds was performed using 2 mM TCEP in 10 mM ammonium bicarbonate at 45 °C for 2 h, followed by alkylation of sulfhydryl bonds at room temperature for 45 min in the dark. Samples were desalted using a Zebra-spin desalting column, and the flow-through was enzymatically digested with trypsin (Promega, Walldorf, Germany) at an enzyme-to-protein ratio of 1:100 and incubated at 37 °C for 4 h. The resulting peptides were reconstituted in 0.1% formic acid and transferred to TruView LCMS vials (Waters, UK) for subsequent analysis.

### 4.7. LC–MS/MS Configuration and Data Processing for Proteomics Analysis

Tryptic peptides were analyzed by liquid chromatography–tandem mass spectrometry (LC–MS/MS). Spectra were acquired in positive ion mode on an Orbitrap HF hybrid mass spectrometer coupled to an EASY-nLC1000 nano-liquid chromatography system (Thermo Fisher Scientific, San Jose, CA, USA) equipped with a nano C18 analytical column, following previously published parameters with minor modifications [100]. Briefly, LC conditions were as follows: Mobile phases A and B were used, with mobile phase A comprising 0.1% formic acid in water and mobile phase B comprising 95% acetonitrile/5% water with 0.1% formic acid. Peptides were directly loaded onto the C18 analytical column and separated by a linear gradient from 2% to 45% B over 135 min at a constant flow rate of 300 nL/min. The column was then washed at 90% B for 10 min and re-equilibrated at 5% B for 35 min. The peptides were analyzed using a data-dependent (TopN15) acquisition method, followed by a higher-energy collisional dissociation at 28% normalized collision energy. Full MS scans were acquired from *m*/*z* 400 to 1600 at a resolution of 120,000 with an AGC target of 3 × 10^6^ ions. MS/MS scans were triggered at an ACG target of 5 × 10^4^ ions and acquired at a resolution of 15,000.

Raw mass spectra were processed using Proteome Discoverer version 2.4 and searched against the reviewed *Homo sapiens* Uniprot protein database (20,434 sequences) [101]. Search parameters were as follows: peptide tolerance, 20 ppm; fragment tolerance, 0.05 Da; minimum fragment ion matches per peptide = 3; digest enzyme = trypsin; fixed modification = cysteine carbamidomethylation; variable modification = methionine oxidation. For quantitative profiling, raw peptide abundances and global intensities were normalized utilizing the median normalization method. The false discovery rate (FDR) for protein identification was set at 1%. Only proteins identified with at least 2 unique peptides were considered for quantitative analysis. Differentially expressed proteins in CBD-, CBG-, and CBN-treated CCA cells were analyzed using Ingenuity Pathway Analysis (IPA). All differentially expressed proteins were uploaded into the IPA core analysis module to identify altered canonical pathways and upstream regulators, as described in a previous study [102]. The IPA database (accessed on 23 April 2023) was used to assess pathway activation or inhibition (*z*-score and adjusted *p*-value). Major signal transduction pathways were reconstructed based on IPA results.

### 4.8. Comparative Pathway Analysis and Hierarchical Clustering

Comparative pathway analysis was performed using Ingenuity Pathway Analysis (IPA, QIAGEN Inc., Germantown, MD, USA). Canonical pathway activation *z*-scores generated from each treatment group (CBD, CBG, CBN, cisplatin, and gefitinib) were exported and used for inter-compound comparison. Similarity among treatments was evaluated by unsupervised hierarchical clustering using Euclidean distance and complete linkage based on IPA-derived pathway activation *z*-scores. Because the clustering was performed using pathway-level activation profiles rather than protein abundance or direct drug–target interactions, cluster proximity reflects similarities in predicted downstream biological responses rather than mechanistic equivalence or shared primary molecular targets.

### 4.9. Statistical Analysis

Results were expressed as mean ± standard deviation (SD) from at least three independent experiments. Statistical comparisons between control and treatment groups were performed using the Student *t*-test, and *p* < 0.05 was considered statistically significant. For proteomic analysis, changes in protein abundance were assessed through pairwise comparisons, and statistical significance was determined using ANOVA with a background-based calculation approach. Adjusted *p*-values were obtained using the Benjamini–Hochberg method to control the FDR. For IPA, upstream regulators were considered significant when they exhibited a *z*-score of ≥1.5 and adjusted *p*-value of <0.05.

## 5. Conclusions

CBD, CBG, and CBN exhibited significant anti-cholangiocarcinoma activity by reducing cell viability, suppressing proliferation, decreasing nuclear Ki67 expression, disrupting mitochondrial membrane potential, and inducing apoptosis in KKU-452 cells. Comparative proteomic profiling coupled with Ingenuity Pathway Analysis (IPA) identified common candidate upstream regulators, including LARP1, TFEB, and the B-cell receptor (BCR) signaling complex, together with distinct pathway-response profiles among the three phytocannabinoids. Hierarchical clustering based on IPA-derived pathway activation *z*-scores demonstrated pathway-level convergence, with CBN exhibiting downstream biological responses more similar to those of cisplatin and gefitinib, whereas CBD and CBG displayed closely related response profiles. These observations should be interpreted as similarities in predicted downstream cellular responses rather than shared molecular mechanisms. Accordingly, the identified upstream regulators represent candidate molecular mediators and potential therapeutic targets that require further functional validation. Overall, this study provides a systems-level proteomic framework for understanding cannabinoid-induced biological responses in cholangiocarcinoma and supports future investigations using targeted molecular approaches, in vivo models, and combination strategies with established anticancer agents before clinical translation.

## Figures and Tables

**Table 1:** IC_50_ values of cannabinoids and reference drugs in KKU-100 and KKU-452 cells.

Compounds	KKU-452		KKU-100	
	24 h	48 h	24 h	48 h
	IC_50_ (µM)	IC_50_ (µM)	IC_50_ (µM)	IC_50_ (µM)
CBD	4.49 ± 1.34	0.58 ± 0.18	6.11 ± 3.83	2.34 ± 0.97
CBG	3.51 ± 1.40	0.54 ± 0.07	4.60 ± 2.06	2.14 ± 0.96
CBN	4.12 ± 1.32	0.50 ± 0.10	7.20 ± 4.50	3.70 ± 1.90
Cisplatin	38.8 ± 13.9	0.66 ± 0.40	65.71 ± 37.43	19.23 ± 10.04
Gefitinib	3.97 ± 1.65	1.27 ± 0.57	45.61 ± 36.01	0.82 ± 0.44

**Table 2:** Top predicted upstream regulators identified by Ingenuity Pathway Analysis (IPA) in KKU-452 cells following treatment with cannabinoids, cisplatin, and gefitinib. Activation state was predicted by IPA based on the proteomic dataset. Positive *z*-scores indicate predicted activation, whereas negative *z*-scores indicate predicted inhibition. The *p*-value of overlap indicates the statistical significance of the overlap between dataset proteins and genes regulated by the upstream regulator.

Upstream Regulator	Treatment	*z*-Score	*p*-Value of Overlap	Target Molecules	Predicted State
LARP1	Gefitinib	−6.940	$1.05\times 10^{-26}$	PABPC1, EIF4G1, EIF4E, RPS6, RPL11, RPL12, RPL13, RPS3	Inhibited
	Cisplatin	−7.162	$3.12\times 10^{-28}$		Inhibited
	CBD	−6.273	$7.54\times 10^{-22}$		Inhibited
	CBG	−6.718	$5.12\times 10^{-24}$		Inhibited
	CBN	−6.940	$2.15\times 10^{-25}$		Inhibited
TFEB	Gefitinib	−4.143	$1.14\times 10^{-12}$	LAMP1, CTSD, CTSB, ATP6V1A, SQSTM1, MAP1LC3B	Inhibited
	Cisplatin	−2.380	$3.45\times 10^{-8}$		Inhibited
	CBD	−3.830	$2.88\times 10^{-11}$		Inhibited
	CBG	−3.080	$6.14\times 10^{-9}$		Inhibited
	CBN	−1.214	$4.21\times 10^{-4}$		No designatable state
BCR (complex)	Gefitinib	−0.655	$4.12\times 10^{-4}$	AKT1, MAPK1, MAPK3, NFKB1, STAT3, CCND1, JUN	No designatable state
	Cisplatin	−0.873	$8.51\times 10^{-5}$		No designatable state
	CBD	−3.183	$1.24\times 10^{-9}$		Inhibited
	CBG	−3.055	$3.56\times 10^{-8}$		Inhibited
	CBN	−1.528	$1.02\times 10^{-5}$		No designatable state
YAP1	Gefitinib	−1.313	$3.55\times 10^{-5}$	CTGF, CYR61, BIRC5, MYC, CCND1, MCL1, AXIN2	No designatable state
	Cisplatin	0.132	$5.18\times 10^{-3}$		No designatable state
	CBD	−2.546	$4.12\times 10^{-7}$		Inhibited
	CBG	−0.694	$1.88\times 10^{-4}$		No designatable state
	CBN	0.165	$2.45\times 10^{-3}$		No designatable state
MASTL	Gefitinib	−1.713	$2.14\times 10^{-6}$	ENSA, ARPP19, CDK1, CCNB1, CDC25C	No designatable state
	Cisplatin	−1.544	$8.95\times 10^{-6}$		No designatable state
	CBD	−2.186	$3.45\times 10^{-8}$		Inhibited
	CBG	−1.943	$1.12\times 10^{-7}$		No designatable state
	CBN	−1.067	$5.64\times 10^{-5}$		No designatable state
CDK4/6	Gefitinib	−2.160	$8.44\times 10^{-8}$	RB1, CCND1, E2F1, MCM2, MCM3, MCM4, PCNA	Inhibited
	Cisplatin	−1.372	$5.12\times 10^{-5}$		No designatable state
	CBD	−1.067	$3.11\times 10^{-4}$		No designatable state
	CBG	−1.372	$4.25\times 10^{-5}$		No designatable state
	CBN	−1.982	$1.08\times 10^{-6}$		No designatable state
MYC	Gefitinib	−0.451	$1.12\times 10^{-3}$	CCND1, CDK4, NPM1, ENO1, LDHA, SFN, NCL	No designatable state
	Cisplatin	0.544	$4.55\times 10^{-2}$		No designatable state
	CBD	−2.176	$5.22\times 10^{-6}$		Inhibited
	CBG	0.108	$3.14\times 10^{-2}$		No designatable state
	CBN	1.085	$1.05\times 10^{-3}$		No designatable state

## Data Availability

The data presented in this study are available in the article and Supplementary Materials.

## Supplementary Materials

The following supporting information can be downloaded at: https://www.mdpi.com/article/10.3390/molecules31142446/s1, The raw measurements are available in Supplementary Files S1–S4. These files include complete data on protein expression profiles, proteomics assessments, microRNA biogenesis signaling pathways, and comparisons of upstream signaling pathways. The Supplementary Materials support the findings presented in Figure 5, Figure 6 and Figure 7.
